# Paired Tumor and Normal Whole Genome Sequencing of Metastatic Olfactory Neuroblastoma

**DOI:** 10.1371/journal.pone.0037029

**Published:** 2012-05-23

**Authors:** Glen J. Weiss, Winnie S. Liang, Tyler Izatt, Shilpi Arora, Irene Cherni, Robert N. Raju, Galen Hostetter, Ahmet Kurdoglu, Alexis Christoforides, Shripad Sinari, Angela S. Baker, Raghu Metpally, Waibhav D. Tembe, Lori Phillips, Daniel D. Von Hoff, David W. Craig, John D. Carpten

**Affiliations:** 1 Virginia G. Piper Cancer Center Clinical Trials at Scottsdale Healthcare (VGPCC), Scottsdale, Arizona, United States of America; 2 Cancer and Cell Biology Division, The Translational Genomics Research Institute, Phoenix, Arizona, United States of America; 3 Collaborative Sequencing Center, The Translational Genomics Research Institute, Phoenix, Arizona, United States of America; 4 Neurogenomics Division, The Translational Genomics Research Institute, Phoenix, Arizona, United States of America; 5 Innovation Center, Kettering, Ohio, United States of America; 6 Integrated Cancer Genomics Division, The Translational Genomics Research Institute, Phoenix, Arizona, United States of America; 7 Collaborative Bioinformatics Center, The Translational Genomics Research Institute, Phoenix, Arizona, United States of America; 8 Clinical Translational Research Division, The Translational Genomics Research Institute, Phoenix, Arizona, United States of America; Howard University, United States of America

## Abstract

**Background:**

Olfactory neuroblastoma (ONB) is a rare cancer of the sinonasal tract with little molecular characterization. We performed whole genome sequencing (WGS) on paired normal and tumor DNA from a patient with metastatic-ONB to identify the somatic alterations that might be drivers of tumorigenesis and/or metastatic progression.

**Methodology/Principal Findings:**

Genomic DNA was isolated from fresh frozen tissue from a metastatic lesion and whole blood, followed by WGS at >30X depth, alignment and mapping, and mutation analyses. Sanger sequencing was used to confirm selected mutations. Sixty-two somatic short nucleotide variants (SNVs) and five deletions were identified inside coding regions, each causing a non-synonymous DNA sequence change. We selected seven SNVs and validated them by Sanger sequencing. In the metastatic ONB samples collected several months prior to WGS, all seven mutations were present. However, in the original surgical resection specimen (prior to evidence of metastatic disease), mutations in *KDR, MYC, SIN3B*, and *NLRC4* genes were not present, suggesting that these were acquired with disease progression and/or as a result of post-treatment effects.

**Conclusions/Significance:**

This work provides insight into the evolution of ONB cancer cells and provides a window into the more complex factors, including tumor clonality and multiple driver mutations.

## Introduction

Previously called esthesioneuroblastoma, olfactory neuroblastoma (ONB) is a rare cancer comprising 2% of all sinonasal tract tumors with an incidence of 0.4 cases per million [1]. ONB is thought to arise from sensory neuroepithelial olfactory cells typically found in the upper portion of the naval cavity [1]. These tumors do not have a gender predilection and can occur at any age, but have a bimodal age distribution in the 2^nd^ and 6^th^ decades of life [1]. The most common presenting symptoms include unilateral nasal obstruction (70%), and epistaxis (50%). Anosmia is not a common complaint (5%) [1]. ONB is a malignant tumor and ∼25% of the patients develop cervical lymph node metastasis [2]. Based on pathology, distinguishing features of ONB include nesting, neurofibrillary stroma and presence of stippled nuclei. Its distinct immunoprofile includes loss of keratin expression, immunopositivity for neuroendocrine markers, and S100 positive cells surrounding the nests of tumor cells. Despite all these distinguishing features, the wide variability in these tumors can lead to difficulty in diagnosis [3]. Surgery and radiation with or without chemotherapy are considered the standard of care for non-distant metastatic disease based primarily on retrospective series [4]. While no standard chemotherapy exists for ONB, cisplatin and etoposide or doxorubicin, or vincristine with an alkylating agent are most commonly administered [5]. However, after such treatment ONB often recurs [6].

Due to the rarity of this disease, most of the published literature on ONB includes case reports or retrospective analysis of ONB patients to predict treatment outcome. There have been very few studies on the molecular characterization of ONB. Expression of Bcl-2, Trk-A and B, Grp78 and several other markers has been analyzed by immunohistochemistry by different groups [7], [8]. Array comparative genomic hybridization (aCGH) has revealed multiple chromosomal aberrations in this tumor type [9]. The study by Guled *et al.* analyzed 13 ONB samples and revealed copy number changes including gains at 7q11.22-q21.11, 9p13.3, 13q, 20p/q, and Xp/q, and losses at 2q31.1, 2q33.3, 2q37.1, 6q16.3, 6q21.33, 6q22.1, 22q11.23, 22q12.1, and Xp/q [9]. In addition, the Hedgehog signaling pathway has been posited to be crucial for ONB development [6]. A study by Koschny *et al.* showed that primary ONB cells are TRAIL (TNF related apoptosis inducing ligand) resistant but are sensitized to TRAIL-induced apoptosis by the proteasome inhibitor bortezomib. This sensitizing effect involves several regulators of the TRAIL signaling pathway. Both these anti-cancer agents are already in clinical use but their effect on ONB patients remain to be evaluated [10]. Sequencing analyses have identified new genes and pathways that have not been previously linked to human cancer [11], [12]. Apart from these studies there is little information on the genomic alterations or changes in cellular signaling in ONB patients. Thus, so far there has been no study to identify mutation profiles of these rare cancers in order to identify new therapeutic targets for treating these patients.

It is universally accepted that somatic alterations (*i.e*., point mutations, small insertions and deletions, rearrangements, gains and losses) occur at the DNA level in cancer. These somatic events can drive tumorigenesis, metastatic progression, and/or drug resistance [13]. More importantly, specific somatic alterations are intimately tied to companion targeted therapeutics. Technological advances have been rapid and in 2007, the first genome sequence of a single individual was deciphered in only 2 months at a cost of less than $2 million [14]. Researchers from the Wellcome Trust in UK published the entire genome of two cancer cell lines [15], [16]. These studies of a malignant melanoma and a lung cancer line, respectively, revealed for the first time almost all of the mutations in the genomes of these two cancers [15], [16]. In order to take full advantage of these technological advances, we have applied such capabilities in the clinic and translated them to the management of disease in individual patients. Currently, physicians have few choices when formulating a treatment plan for a patient with advanced cancer especially in case of rare cancers, as there is usually very little published literature about these diseases. Thus, we are far from understanding the genetics and disease progression in these diseases. As demonstrated, whole genome sequencing (WGS) technologies now provide us with platforms to interrogate entire human genomes at a fraction of the time and cost compared to more traditional sequencing technologies. These technologies, for the first time, offer us the ability to survey the global somatic landscape of cancer. It is now possible with WGS to re-sequence, analyze, and compare the matched tumor and normal genes of an individual patient. With these paradigm shifts in technologic capabilities, we present the results of the first paired tumor and normal whole genome sequences of metastatic ONB.

## Methods

### Ethics Statement

The study was approved by the Western Institutional Review Board (WIRB) and was conducted in accordance with the 1996 Declaration of Helsinki. This was a pilot study entitled, “An Ancillary Pilot Trial Using Whole Genome Tumor Sequencing in Patients with Advanced Refractory Cancer” (WIRB® Protocol #20101288)(NCT01443390). Informed consent was obtained from the patient with olfactory neuroblastoma, including written consent for publication of the clinical details and images.

### Eligibility Criteria

Patients of age ≥18 that provided signed informed consent, with relapsed or refractory solid tumors, who progressed on at least one systemic therapy for advanced disease and willing to undergo a biopsy or surgical procedure to obtain tissue, which may not be a part of the patient’s routine care for their malignancy were eligible for the study. Other eligibility criteria included: Karnofsky performance status (PS) ≥80%, life expectancy >3 months, baseline laboratory data indicating acceptable bone marrow reserve, liver, and renal function. Patients were allowed to participate on another clinical trial involving treatment prior to or during participation on this study. Main exclusion criteria included: symptomatic central nervous system (CNS) metastasis, untreated CNS metastases, known active infections requiring intravenous antimicrobial therapy, known HIV, HBV or HCV infection requiring antiviral therapy, pregnant or breast feeding women, or inaccessible tumor for biopsy. While additional patients were recruited under this protocol to perform WGS of both their tumor DNA and germline DNA, no other patient had a diagnosis of ONB or head and neck cancer.

### Case Report

A 29-year-old man with metastatic ONB presented to Virginia G. Piper Cancer Center Clinical Trials at Scottsdale Healthcare for participation in a pilot study to apply WGS on a fresh biopsy of one of his metastatic lesions to determine if identification of somatic perturbations might be useful for downstream therapy. The initial diagnosis of ONB was made after expert pathologic review at a major academic center. The tumor consisted of nests of closely packed cells with small to medium sized nuclei and scant cytoplasm. Within the nests, single cell and occasional necrosis was present. The tumor had features of epithelial differentiation confirmed by pankeratin staining, which highlights the clusters and single cells within the nests with strong cytoplasmic staining. The rest of the cells stain strongly for synaptophysin supporting the diagnosis of high grade ONB. The tumor was negative for chromogranin, neurofilament, CD45, CD20, S100, HMB45, and Melan A. Subsequently collected specimens also underwent confirmatory pathology review for the ONB diagnosis.

### Sample Assessment

Samples were received for tumor confirmation, analyte (DNA) extraction and processing for downstream WGS experiments. All tumor samples were obtained under IRB approved protocol, were preserved as fresh frozen and reference DNA was obtained from peripheral blood mononuclear cells. Samples from the patient with ONB were collected at SHC (Scottsdale Healthcare, Scottsdale, AZ) under radiographic guidance, flash frozen in liquid nitrogen and transported to TGen (The Translational Genomics Research Institute, Phoenix, AZ) on dry ice and stored at −80°C until sample processing. Direct visualization of samples collected was obtained by two-ink frozen quality control (QC) procedure to estimate tumor content and extent of tissue heterogeneity by a board-certified pathologist (GH). All tumor samples used had greater than 50% tumor content. Digital image files of all QC tissues were scanned using Aperio GL scanner and image files were stored on secure web-based viewing by Spectrum Plus (Aperio, Inc).

### Genomic DNA Isolation

#### Fresh frozen tissue

Tissue collected from ultrasound-guided 18-gauge needle biopsy was disrupted and homogenized in Buffer RLT plus, Qiagen AllPrep DNA/RNA Mini Kit, using the Bullet BlenderTM, Next Advance. Specifically, approximately 7 mg of tissue was transferred to a microcentrifuge tube containing 600 µl of Buffer RLT plus, and 9 mg of 1.6 mm stainless steel beads. The tissue was homogenized in the Bullet Blender at room temperature at Speed 10 for 5 minutes. The sample was centrifuged at full speed and the lysate was transferred to the Qiagen AllPrep DNA spin column. Genomic DNA purification was conducted as directed by the AllPrep DNA/RNA Mini Handbook, Qiagen. DNA was quantified by Nanodrop spectrophotometer for appropriate dilution and quality was accessed using 260/280 and 260/230 absorbance ratios.

#### Whole blood

Genomic DNA was isolated from frozen blood using the Qiagen QiaAmp DNA Blood Midi Kit. Specifically, blood (12 ml) that was previously frozen and stored at −80°C was allowed to equilibrate to room temperature. Two ml of blood was transferred to conical tubes and treated with protease. Each 2 ml aliquot was then applied to Qiagen QiaAmp Midi columns and genomic DNA was purified as directed by the Qiagen QiaAmp DNA Blood Midi/Maxi Handbook. DNA was quantified using the Nanodrop spectrophotometer and quality was accessed from 260/280 and 260/230 absorbance ratios.

#### Whole genome library preparation

3 µg of genomic DNA from each sample (control and tumor) was used for library preparation. Samples were prepared for sequencing using proprietary methods. In summary, samples were fragmented using the Covaris S2 system (part#4387833) to a target fragment size of 300 to 350 base pairs (bp). Fragmentation was verified by running samples on a 2% Tris Acetate EDTA (TAE) gel. Overhangs in the fragmented samples were then repaired to form blunt ends using T4 DNA polymerase and Klenow (New England Biolabs; NebNext DNA Sample Prep Master Mix Set I; catalog # E6040L), and products were cleaned using Agencourt Ampure magnetic beads (Beckman Coulter Genomics; catalog # A29153). Adenine bases were next ligated onto the blunted fragments using Klenow exo (NebNext DNA Sample Prep Master Mix Set I), and A-tailed products were cleaned using Ampure magnetic beads. Products were next quantified using Quant-iT Picogreen dsDNA reagent (Invitrogen; catalog # P11496) in triplicate with a 0 ng/µL to 200 ng/µL standard. To prepare for adaptor ligation, samples were vacuum dried to the appropriate volume to allow for a 10∶1 adaptor to DNA molar ratio. Diluted paired end Illumina adapters were then ligated onto the A-tailed products using DNA ligase (NebNext DNA Sample Prep Master Mix Set I). Following ligation, samples were run on a 3% TAE gel at 120V for 2.5 hours to separate ligated products. X-tracta gel extractors (USA Scientific; catalog # 5454-0100) were used to select ligation products at 300 bp and 350 bp for each sample. Ligated products were isolated from these gel punches using Freeze ‘N Squeeze DNA Gel Extraction Spin Columns (Bio-rad; catalog # 732-6166), and cleaned using Ampure magnetic beads. 2X Phusion High-Fidelity PCR Master Mix (Finnzymes; catalog # F-531L) was used to perform PCR in quadruplicate (10 uL ligation/reaction) to enrich for these products. Enriched PCR products were run on a 2% TAE gel and were selected using xtracta gel extractors. PCR products were purified from gel punches using Freeze ‘N Squeeze DNA Gel Extraction Spin Columns. Extracted products were purified using Ampure magnetic beads and quantified using Agilent’s High Sensitivity DNA chip (catalog # 5067-4626) on the Agilent 2100 Bioanalyzer (catalog # G2939AA).

#### Paired end next generation sequencing

Tumor and normal libraries were prepared for paired end sequencing. Samples were denatured using 2N NaOH and diluted with Illumina HT2 buffer. Clusters were generated on all flowcells using Illumina’s cBot and HiSeq Paired End Cluster Generation Kits. Flowcells were sequenced on Illumina’s HiSeq 2000 using Illumina’s HiSeq SBS Sequencing Kit for two paired end 100 bp sequencing runs per flowcell.

#### Data analysis

The Illumina HiSeq 2000 generated raw sequence data in the form of .bcl files. These data were converted to .qseq files using Illumina’s BCL Converter tool, and resulting .qseq files were used to generate .fastq files for downstream analysis. Fastq files were validated to evaluate the distribution of quality scores and to ensure that quality scores do not drastically drop over each read. Validated fastq files were aligned to the human reference genome (build 36) using the Burrows-Wheeler Alignment (bwa) tool [17], which uses the Burrows-Wheeler Transform (BWT) algorithm. Following alignment, generated .sai files were used to create .sam (sequence alignment map) files by converting suffix array coordinates to chromosomal coordinates [17]. Resulting .sam files were input into SAMtools [18] to create binary sequence (.bam) files. PCR duplicates were flagged for removal using Picard (http://picard.sourceforge.net), and base quality scores were recalibrated using GATK (Genome Analysis Toolkit) [19]. Mutation analysis was performed to identify single nucleotide polymorphisms (SNPs), insertion/deletions (indels), and copy number variants. Results from all mutation analyses are summarized in a Circos plot [20].

#### Single nucleotide variant (SNV) identification

SNP calling was performed using SolSNP (http://sourceforge.net/projects/solsnp/files/SolSNP-1.01/) and Mutation Walker, a tool developed in house and that incorporates variant discovery tools from GATK. SNPs that were called using both tools were compiled for further examination. Two sets of thresholds, strict and lenient, were enabled to reduce the false negative rate. Data from each of these two sets were visually examined for false positives to create a final filtered list of true SNVs, which were annotated with GENCODE using an internal annotation script.

SolSNP is an individual sample mutation detector implemented in Java. The algorithm is based on modified Kolmogorov-Smirnov like statistics, which incorporates base quality scores. The algorithm is non-parametric and makes no assumptions on the nature of the data. It compares the discrete sampled distribution, the pileup on each strand, to the expected distributions (according to ploidy). In the case of a diploid genome, both strands need to provide evidence for the variation. Zero quality bases are trimmed off the pileup before comparison. SolSNP is a standalone program that can be run from the command line and is general enough to work with any next generation sequencing data with high coverage. While making somatic calls, SolSNP’s high quality genotype call is made for all callable loci of the normal sample. To reduce false negatives, variant loci in tumor samples are called with the Variant Consensus mode. Variant loci in tumor samples that exhibit a high quality homozygous reference genotype in the normal sample are considered as somatic. To call somatic variants, SolSNP is augmented by a Python script.

#### Insertion/deletion identification

Indel calling was performed using GATK and a somatic indel detection tool developed in house. For detecting somatic indels we employed a two-step strategy. In the first step, we removed reads whose insert size laid outside the interval (50,500) from the tumor bam files. GATK was then used to generate a list of potential small indels from this bam. A customized Perl script, which uses the Bio-SamTools library from BioPerl [21], takes these indel positions and for each of the indels, looks at the region in the normal sample that is 5 bases upstream from the start of the indel and 5 bases downstream from the end of the indel. An indel was determined to be somatic only if there was no indel detected in the region under consideration.

#### Copy number analysis

Copy number variants were identified by an analysis of differential clone coverage. In this assessment, a single fragment includes the two sequenced paired ends and the unsequenced interlying region as being covered by one clone. Unmapped reads and reads lying within 2 standard deviations outside of the insert distance are not included. Relative copy number is determined as the log2 difference between the normal and tumor normalized coverage, where normalization is the mean coverage across a 2 kb window divided by the genome-wide mode coverage.

### Validation of Mutations Identified by WGS

#### DNA extraction from the formalin-fixed, paraffin-embedded (FFPE) tissue sections

FFPE blocks were obtained under an approved IRB protocol. H&E sections were prepared from each patient block and areas of tumor cells were marked. A 12 µm section was then cut for each patient block and respective H&E sections were overlaid to identify areas of tumor cell enrichment. Next, the tissue sections were deparaffinized and areas of tumor cells were scraped and DNA extractions were performed using RecoverAll kit from Ambion (Invitrogen, Carlsbad, CA) according to manufacturer’s protocol.

#### Sanger sequencing to confirm mutations

Specific PCR primers were designed for the genomic sequences for each of the mutated genes chosen for further validation. Primers were designed such that PCR products ranged from 200–350 bp. Primer sequences for the seven genes chosen for the validation of mutations are included in Table S1. M13 sequences were included in the forward and reverse primers specific to each gene as a back-up for sequencing reactions. DNA was extracted from each of the samples as described above and 10 ng DNA was used for each specific PCR reaction. Platinum Taq high fidelity DNA polymerase (Invitrogen Inc., Carlsbad, CA) was used for the PCR and the reactions were performed according to manufacturer’s protocols. PCR was run for 35 cycles of: Denaturation: 94°C for 20 sec, Annealing: 58.5°C for 30 sec and elongation: 68°C for 30 sec. Upon completion, PCR products were purified using QIAquick PCR purification kit (Qiagen Inc., Valencia, CA) and sent for PCR sequencing to Arizona State University DNA sequencing Core facility. All the reactions were forward and reverse sequenced using specific forward and reverse primers. In a few cases, M13 primers were also utilized for the sequencing reactions.

## Results

### Case Report

A 29-year-old man with metastatic ONB presented to Virginia G. Piper Cancer Center Clinical Trials at Scottsdale Healthcare for participation in a pilot study to apply WGS on a fresh biopsy of one of his metastatic lesions to determine if identification of somatic perturbations might be useful for downstream therapy.

Prior to the cancer diagnosis, his past medical history was significant for obesity. Pertinent past environmental exposure history included tobacco chewing for five years, and chemical or fume exposures in the work place (tool/dye shop). In early 2008, he presented with symptoms of sinus congestion, sore throat, headache, and unexplained vomiting. Evaluation and imaging at another institution revealed a 4.2 cm mass in the nasal cavity extending into the ethmoid and frontal sinuses. Biopsy confirmed a diagnosis of ONB. He underwent surgery involving both head and neck and neurosurgery consisting of an anterior craniofacial resection, sphenoidectomy, total ethmoidectomy, and transnasal craniotomy in June 2008. He then received radiation therapy 66.6 Gy over 37 fractions from June to September 2008 with concurrent carboplatin and vincristine. After radiotherapy, consolidation carboplatin and vincristine chemotherapy was delivered through March 2009.

In June 2009, he underwent a dacryocystorhinostomy for epiphora, and showed no evidence of cancer. Subsequently, he had development of several local regional recurrences including nasal bridge recurrence in December 2009. He underwent extensive skull base surgery at the end of January 2010 including neck nodal dissection. One of 21 lymph nodes had microscopic metastatic disease and he had tumor that was extending to the ethmoid region to the orbital wall. After undergoing plastic reconstruction with a right forearm free flap, cranioplasty and skin grafting, radiation therapy (total dose of 54 Gy) was delivered to the right neck from March 2010 to May 2010. A right cheek metastasis measuring 3×3 cm was confirmed by excisional biopsy in July 2010. He underwent a surgical debridement of the forehead flap in August 2010. In October 2010, tumor recurrence on the left ridge was confirmed by a biopsy and was visible on the bilateral nasal and ocular canthi, and suspected to involve the right parotid.

At presentation to our clinic, he was on a liquid diet but could swallow pills. His main complaint was xerostomia, and he had anosmia, moderate fatigue, and frontal pressure headaches requiring opiate pain medication. His Karnofsky performance status was 80, and he had right-sided exophthalmos limiting his ability to fully close the right eyelid. Pertinent physical exam findings included several visible areas of metastases in the nasal ridge and orbital ridge, and a palpable hard, fixed mass in the right parotid region (Figure S1A photos). Radiographic imaging was conducted at our institution and revealed multiple foci of tumor (Figures S1B-H CT/MRI images).

### Whole Genome Sequencing

**Figure 1:**
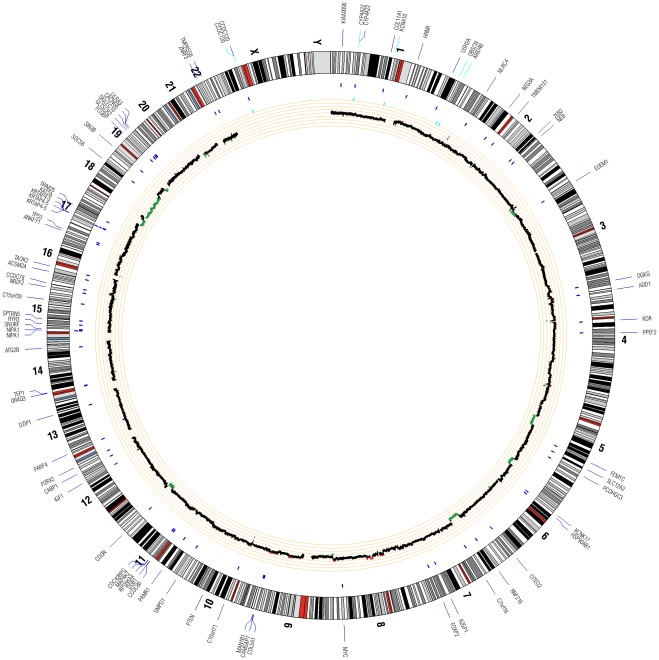
Circos plot for WGS results for ONB. This figure depicts the genomic location in the outer ring and chromosomal copy number in the inner ring. The SNVs and indels are marked on the outer ring in their respective genomic locations. In the inner ring, copy gains are shown in red, while copy losses are shown in green. No interchromosomal translocations were observed by assessing counts of anomalous read pairs between specific regions of the genomes, noting that the use of shorter-paired end sequencing may limit our ability to detect these events with this analysis.

**Figure 2:**
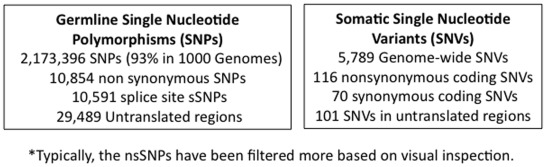
Differences between the germline and somatic sequences. Details the statistics for the germline SNPs and somatic SNVs.

Signed informed consent was obtained and after confirming eligibility, the patient underwent a biopsy in the right parotid region for tumor DNA collection and a venous blood draw for normal germline DNA. WGS was performed after DNA isolation. We generated 463.5GB of total sequencing data with an average Q30 percentage of 81.3% for a total of 379.6GB of Q30 data. We achieved average coverages of 71X for the normal sample and 68X for the tumor. A total of 2,173,398 SNPs were found in the germline with 87% existing in dbSNP and a 2.1 transition/transversion rate. A total of 5,789 SNVs were found across the genome. Of these, sixty-two somatic non-synonymous or missense SNVs and 5 coding indels were identified inside coding regions–these events each cause a non-synonymous change in the DNA sequence as shown in the Circos plot [20] (Figure 1, 2 and Table S2). These indels were a base deletion of *CYP4A22* (1:47384338 del T), a seven base insertion in *KCNA10* (1:110861798 ins GAGCAAC), an insertion of 1 bp in *OBSCN* (1:22657241 ins T), a 1 base deletion in *ARID4B* (1:233411716 Del A), and 7 base deletion in *CCDC120* (X:48811750 del TCGTAGC). A total of 119 genes were found to be somatically lost resulting from a near complete single copy deletion of chromosome 18, focal deletions at 5q15, 6 p25.1, 7p15.3, 7p21.3, 11q24.2, 19p12, and 21q.1. By comparison, a total of 45 genes were found to be gained or amplified resulting from amplification of 8p, focal events at 5p15.33, 7p13, 8q24.3, 9q22.31, 9q34.3, 16q22.1, and 16q24.3. A total of 4/104 coding SNVs were also found in 1000 Genomes database of germline variation, consistent with a less than 5% false positive rate of SNVs actually being germline events that were poorly covered. Frequently mutated genes based on the Cosmic database (Sanger) include *FGFR1* [22], *FANCC*, *NOTCH1* [23], and *CBFA2T3* were all amplified and *JAZF1* was deleted. Other key genes include amplifications of *RXRA*, *NSMAF*, and *ASPH* [24]. Deletions of other key genes include *ETS1* [25], [26], [27], *CCNH*, and coagulation factor *XIII A1* (F13A1).

### Validation of Mutations Identified by WGS

Seven mutated genes namely, *MAP4K2*, *SIN3B*, *TAOK2*, *KDR*, *TP53*, *MYC*, and *NLRC4* were chosen to validate the presence of specific gene mutations as shown in Table 1. This selection was based on clinical relevance and previously published literature on the target genes and their association with carcinogenesis. SIFT results classified all the mutations except *NLRC4*, as damaging. Similar results were seen after Polyphen protein analysis, which classified the mutation in *NLRC4* as benign, while all the others were classified as damaging. For validation, primers were designed for specific gene amplification (Table S1) and the PCR products were Sanger sequenced. Sanger sequencing confirmed mutations in all seven genes (Figures S2A-D) in the patient sample used for WGS. Next, we examined these seven validated mutations in previously collected archival FFPE samples from this ONB patient (Table 2). In the metastatic FFPE samples of the right parotid collected several months prior to WGS, all seven mutations were present. Interestingly, in the original surgical resection specimens (prior to evidence of metastatic disease), mutations in *KDR, MYC, SIN3B*, and *NLRC4 genes* were not present, while *TP53, MAP4K2* and *TAOK2* mutations were present in the original surgical specimen as well.

**Table 1:** Single nucleotide variations in the seven genes chosen for validation by Sanger sequencing.

GENE ID	CHR	Position	Coding	Codon Position	Reference Codon	Reference amino acid	Mutated Codon	Mutated Amino acid	Type	SIFT results	Polyphen results
*MAP4K2*	11	64313974	CDS	761	AGC	S	A**T**C	I	nsSNP	Damaging	Possibly damaging
*TAOK2*	16	29898286	CDS	204	GGC	G	G**A**C	D	nsSNP	Tolerated	Possibly damaging
*TP53*	17	7519128	CDS	176	TGC	C	T**T**C	F	nsSNP	Damaging	Possibly damaging
*SIN3B*	19	16834366	CDS	421	AAG	K	A**T**G	M	nsSNP	Damaging	Possibly damaging
*NLRC4*	2	32329712	CDS	242	AGG	R	A**A**G	K	nsSNP	Tolerated	Benign
*KDR*	4	55671617	CDS	351	CCT	P	C**T**T	L	nsSNP	Damaging	Possibly damaging
*MYC*	8	128820394	CDS	235	CCC	P	C**T**C	L	nsSNP	DamagingLC*	Possibly damaging

Key: *Low confidence, Mutated Codon column-somatic mutation depicted in bold face.

**Table 2:** Validation of mutations in previously collected archival FFPE samples from the ONB patient.

Sample Date	Site examined	*MYC*	*KDR*	*TAOK2*	*MAP4K2*	*SIN3B*	*TP53*	*NLRC4*	Comments
**6/2/08**	Left nasal mass	CCC	AGG	***GAC***	***ATC***	AAG	TTC	AGG	At diagnosis, mutations in *TAOK2/MAP4K2/TP53*
	Right nasal mass	CCC	AGG	***GAC***	***ATC***	AAG	TTC	AGG	
**6/12/08**	Frontal tumor	CCC	AGG	***GAC***	***ATC***	AAG	TTC	AGG	Resection samples do not have new mutations
	Nasal tumor	CCC	N/A	***GAC***	***ATC***	AAG	TTC	AGG	
**7/26/10**	Right cheek	***CTC***	***AAG***	***GAC***	***ATC***	***ATG***	***TTC***	***AAG***	Metastatic samples show evolution of new mutations in *MYC/KDR/SIN3B/NLRC4*
	Right cheek	***CTC***	***AAG***	***GAC***	***ATC***	***ATG***	***TTC***	***AAG***	
**11/3/10**	Right cheek (WGS)	***CTC***	***AAG***	***GAC***	***ATC***	***ATG***	***TTC***	***AAG***	
		CCC	AGG	GGC	AGC	AAG	TGC	AGG	**Reference codons**

Key: Validated mutations compared to reference codon depicted in bold italics.

## Discussion

ONB is a rare cancer of the sinonasal tract and data on its molecular characterization is limited. We performed WGS on paired normal and tumor DNA from a patient with metastatic ONB in an attempt to survey the somatic alterations that might be drivers of tumorigenesis and/or metastatic progression in this disease. After validation of seven selected SNVs in the sequenced tumor sample, we analyzed archival metastatic and primary resection FFPE samples available from the same patient. We found that specific mutations in *KDR*, *MYC*, *SIN3B*, and *NLRC4* appear only in the metastatic setting, while mutations in *TP53*, *TAOK2* and *MAP4K2* were also present in the previous samples (original biopsies of the ONB tumor). There was a mutation in codon 176 in the *p53* gene, which results in a residue change from a cysteine to a phenylalanine. Majority of mutations in *p53* occur in the core domain that contains the sequence-specific DNA binding activity of the p53 protein, and the mutations result in loss of DNA binding activity. The core domain structure consists of two loops (L2 and L3) and the LSH motif and this particular mutation (codon 176) is in the L2 loop [28]. The L2 loop, although not directly involved in DNA binding, is involved in extensive interactions with the L3 loop for binding together zinc atom (Cys176 on the L2 loop is a ligand for the zinc) [29]. In another study, tumors from 26 ONB samples microscopically more closely resembled paragangliomas, and aberrant expression of *TP53* was noted in 62% of cases [30]. Absence of *TP53* mutations in 14 ONB cases with hyper-expression of wild-type *TP53* was found in four metastatic/recurrent cases. One study suggested that *TP53* point mutations did not play an important role in the initial development of ONB as wild-type *TP53* hyper-expression may lead to local aggressive behavior and a tendency for recurrence [31]. Immunohistochemical analysis of one ONB case revealed that 10% of the tumor stained positive for *TP53* protein and vascular endothelial growth factor (VEGF) [32]. This mutation has been previously associated with several other cancer types including oral cancers and Ewing’s sarcoma [29], [33]. In the study involving oral cancers, both the patients with this particular mutation died of the disease within 12 months [29].

Similarly mutations in *MYC* and *KDR*, though not as common as p53 mutations, have been previously described and associated with cancer [34], [35]. Castaneda *et al* have shown N-MYC and MYC expression in a primary ONB sample by northern blot analysis [36]. Several of these genes/their protein products are known to interact/regulate other genes on the list and play important roles in cancer cell signal transduction. For example, MYC binds to SIN3B promoter directly [37]. Similarly, p53 binds to NLRC4 promoter and activates its expression in a cell-type-specific manner [38]. Thus, it would be interesting to study these interactions *in vitro* in cell line model systems in which these genes were mutated.

Our work has identified and validated seven mutations in an ONB patient. In addition, we tested these mutations in additional samples from the same patient. This work provides insight into the evolution of cancer cells and provides a window into the more complex factors at play here, including tumor clonality and multiple driver mutations. Based on these data it is clear that the tumor displays additional mutations. Normal course of disease progression could be responsible for their acquisition. Alternatively, additional mutations could have arisen in response to the primary radiation and/or carboplatin/vincristine chemotherapy as a mechanism of resistance. These mutations may have been present in the subclones of the primary tumor and selected for by dynamic tumor microenvironment. Lastly, as tumors from only one individual were sequenced we do not know which of the SNVs reported here are driver mutations as opposed to passenger mutations occurring by chance. All these hypotheses need to be tested in optimal *in vitro* model systems. Additionally, the mutated target genes and their cellular signaling mechanisms indicate aberrations in DNA repair mechanisms, which may be related to ONB progression in this patient. A limitation of this study is the lack of additional metastastic ONB tumors to assess by WGS. With the reduction in cost, improvement in speed of analysis and with more complete understanding of complex genetic alterations, we anticipate that WGS will be applied in the clinic more frequently to common and rare cancers and will pave the way to personalized medicine.

## Supporting Information

Figure S1 **Images of metastatic olfactory neuroblastoma.**
(PPTX)

Figure S1A Photograph of metastatic olfactory neuroblastoma mass in the right parotid region that was subsequently biopsied for tumor whole genome sequencing.
(TIF)

Figure S1B CT axial image depicting extent of local disease recurrence of the olfactory neuroblastoma. Arrow points to the heterogeneous mass.
(TIF)

Figure S1C and S1D Spin echo T1 weighted with contrast (1C) and FLAIR sequence (1D) MRI axial images depicting extent of local disease recurrence of the olfactory neuroblastoma. Arrow points to the heterogeneous mass.
(TIF)

Figure S1E and S1F Spin echo T1 weighted pre-contrast (1E) and spin echo fast scan (1F) MRI coronal images depicting extent of local disease recurrence of the olfactory neuroblastoma. Arrows point to the heterogeneous mass.
(TIF)

Figure S1G and S1H CT axial image (1G) and MRI coronal spin echo fast scan image (1H) depicting metastasis to the right parotid region. Arrow points to the mass that was biopsied for tumor whole genome sequencing.
(TIF)

Figure S2 **Genomic regions of** ***KDR*** **and** ***MYC*** **genes harboring SNVs.** DNA alignments and sequencing electropherograms depicting specific SNVs in *KDR* (Figures S2A and S2B) and *MYC* (Figures S2C and S2D) genes. Arrows in Figures S2B and S2D point to the mutated residue in the electropherograms. Electropherogram shown for *KDR* (Figure S2B) is for the sequencing reaction of the complementary strand.
(PDF)

Table S1 **Primers used for PCR amplification and sequencing of the mutated regions.** Key: *The sequences in bold are the M13 forward and reverse primer sequences added to our specific primers to aid in sequencing reactions.
(XLS)

Table S2 **Summary of mutated genes, N = 67.**
(XLS)
