# Supplementary material for: Paired Tumor and Normal Whole Genome Sequencing of Metastatic Olfactory Neuroblastoma
Source: PLoS One. 2012 May 23;7(5):e37029. doi: 10.1371/journal.pone.0037029 (PMC3359355; doi:10.1371/journal.pone.0037029)
Supplement: Figure S1 — Images of metastatic olfactory neuroblastoma. (PPTX) [file pone.0037029.s001.pptx]

## Slide 1
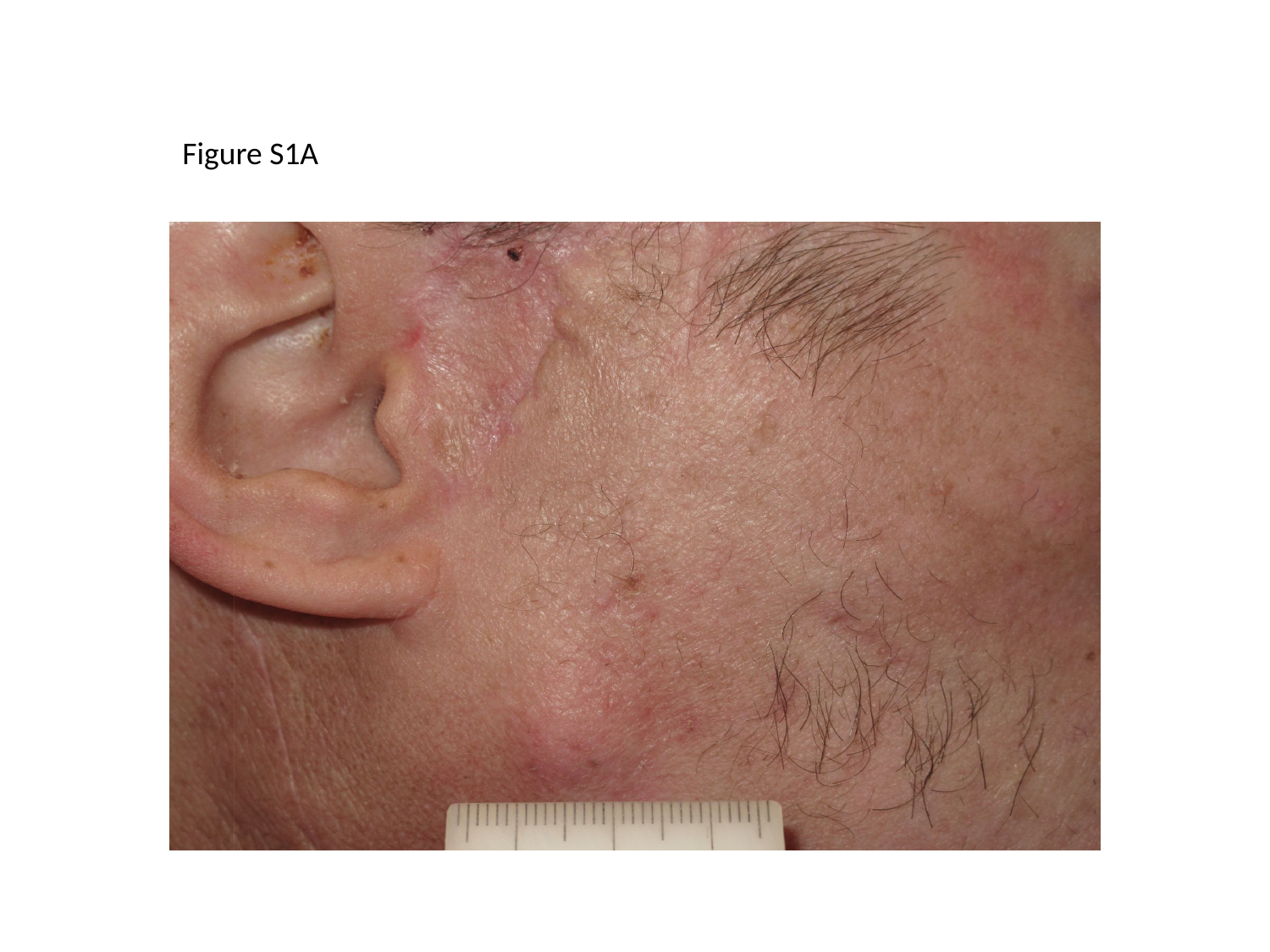

Figure S1A

## Slide 2
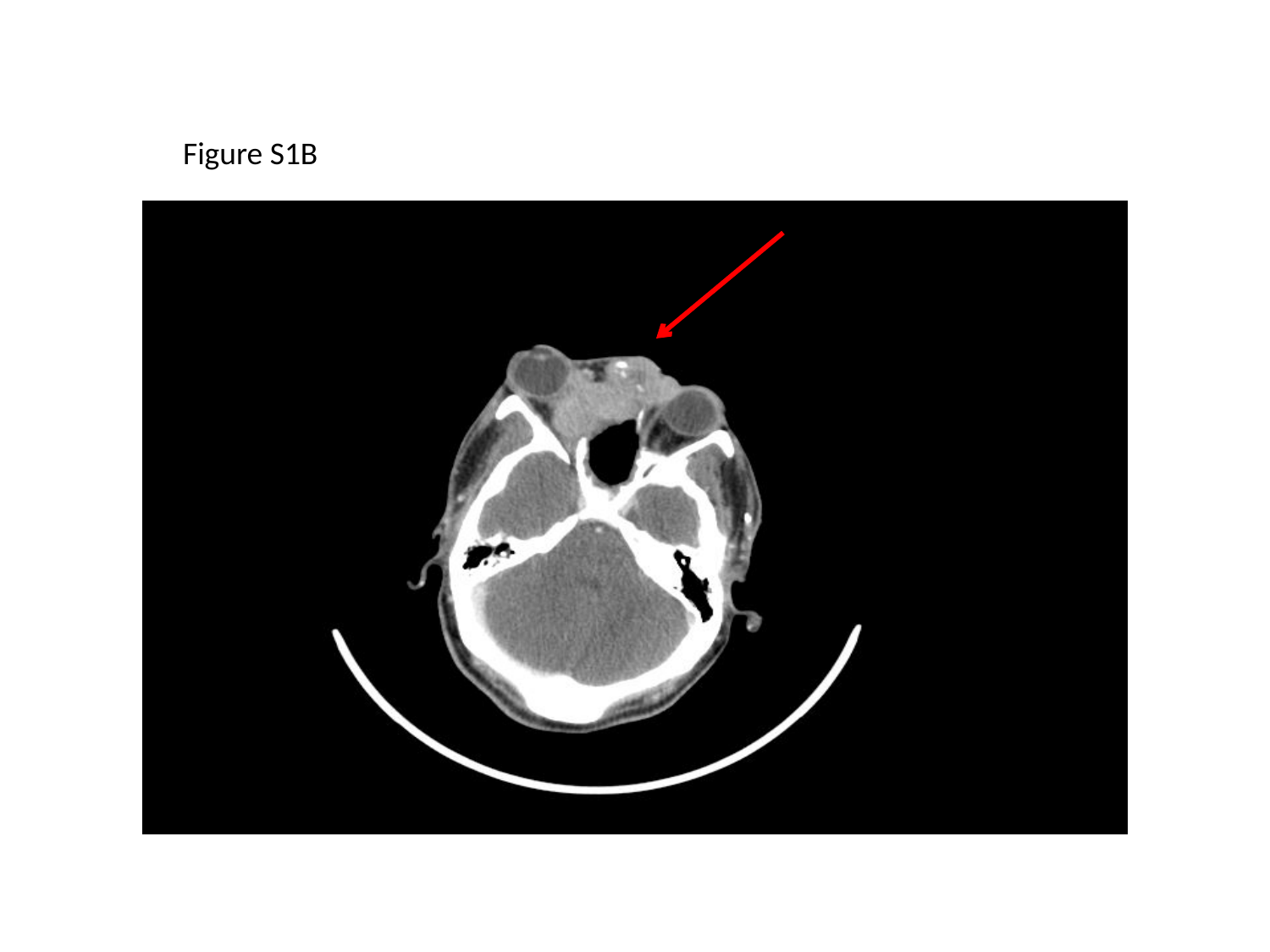

Figure S1B

## Slide 3
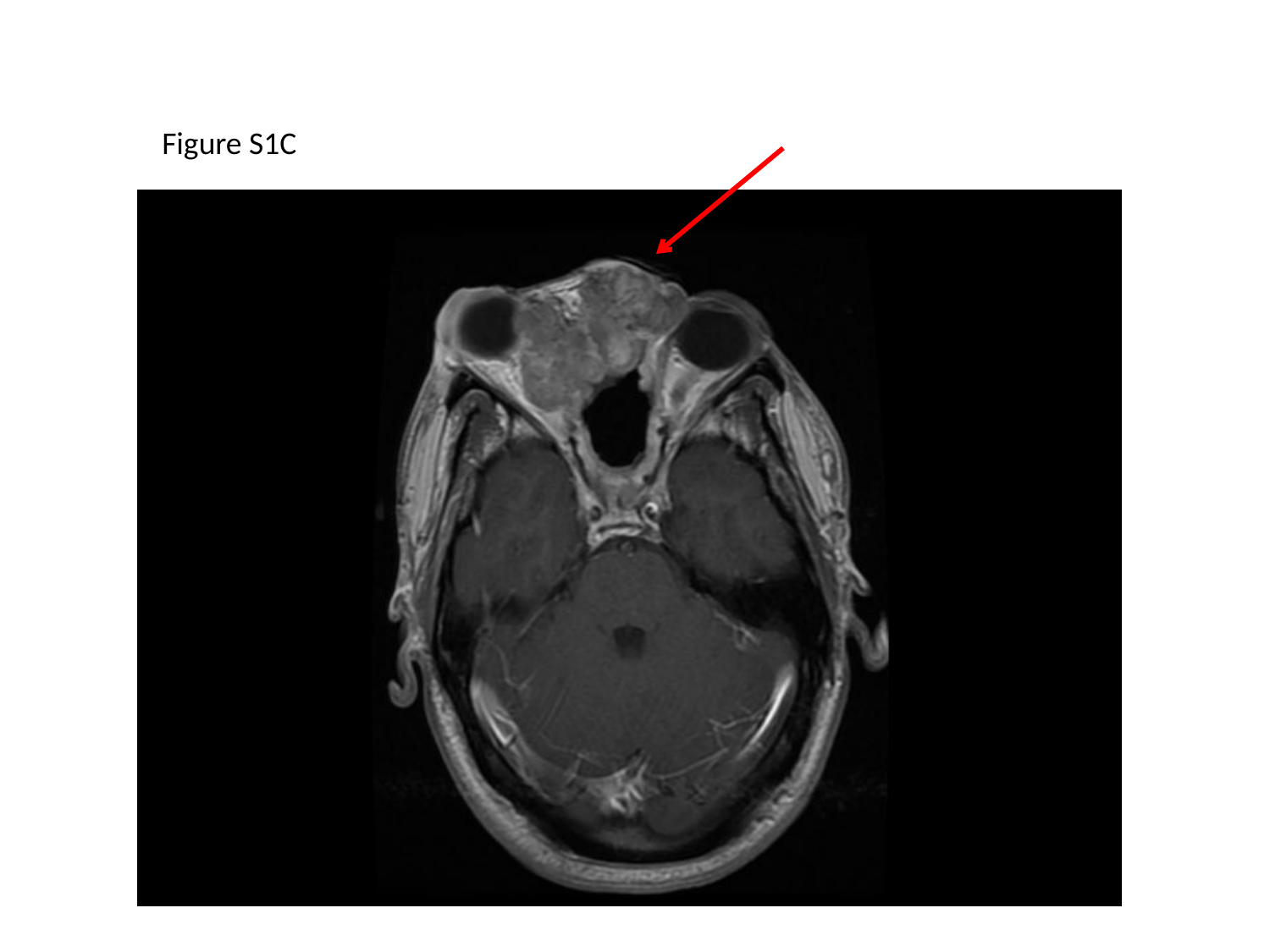

Figure S1C

## Slide 4
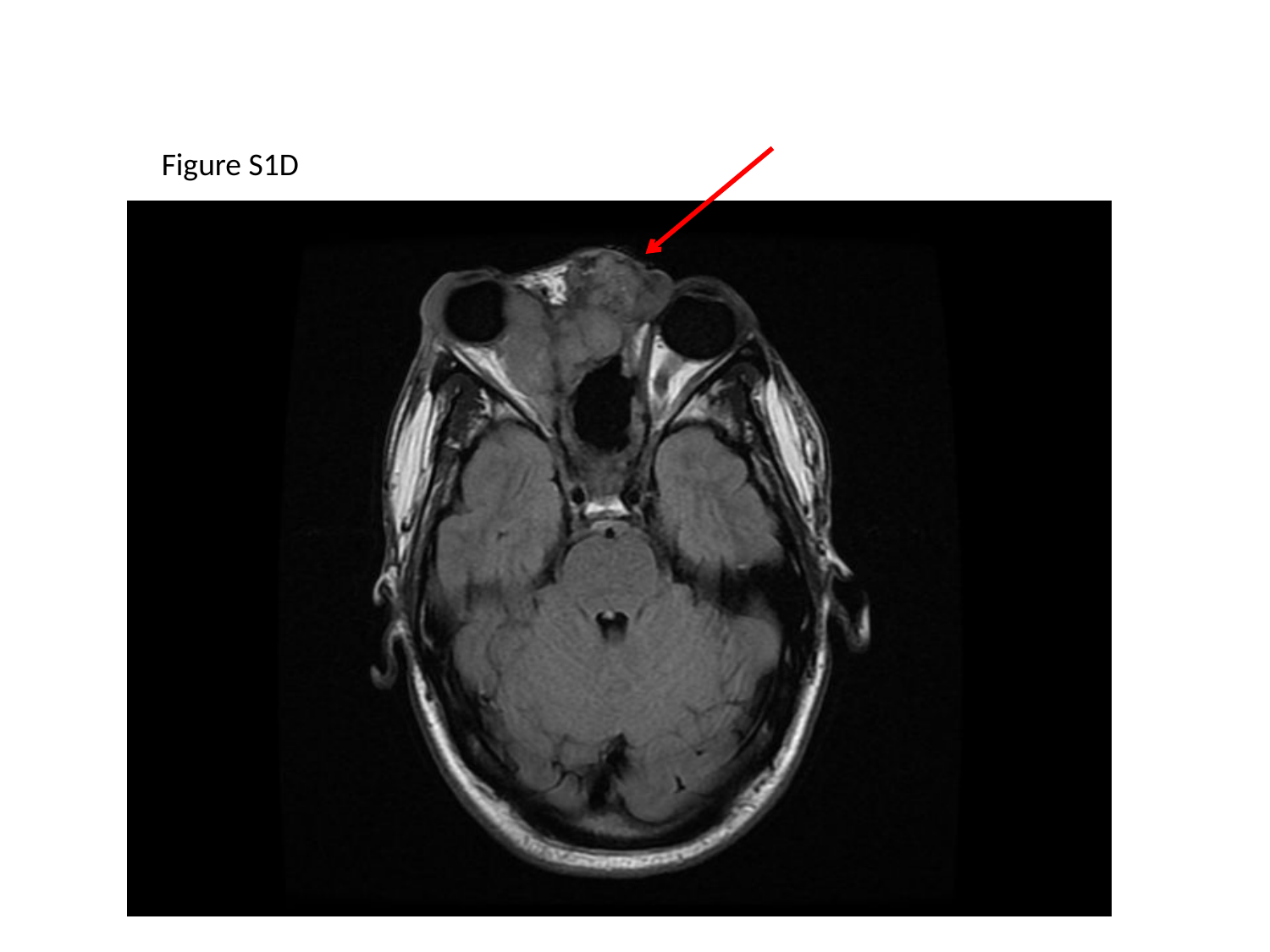

Figure S1D

## Slide 5
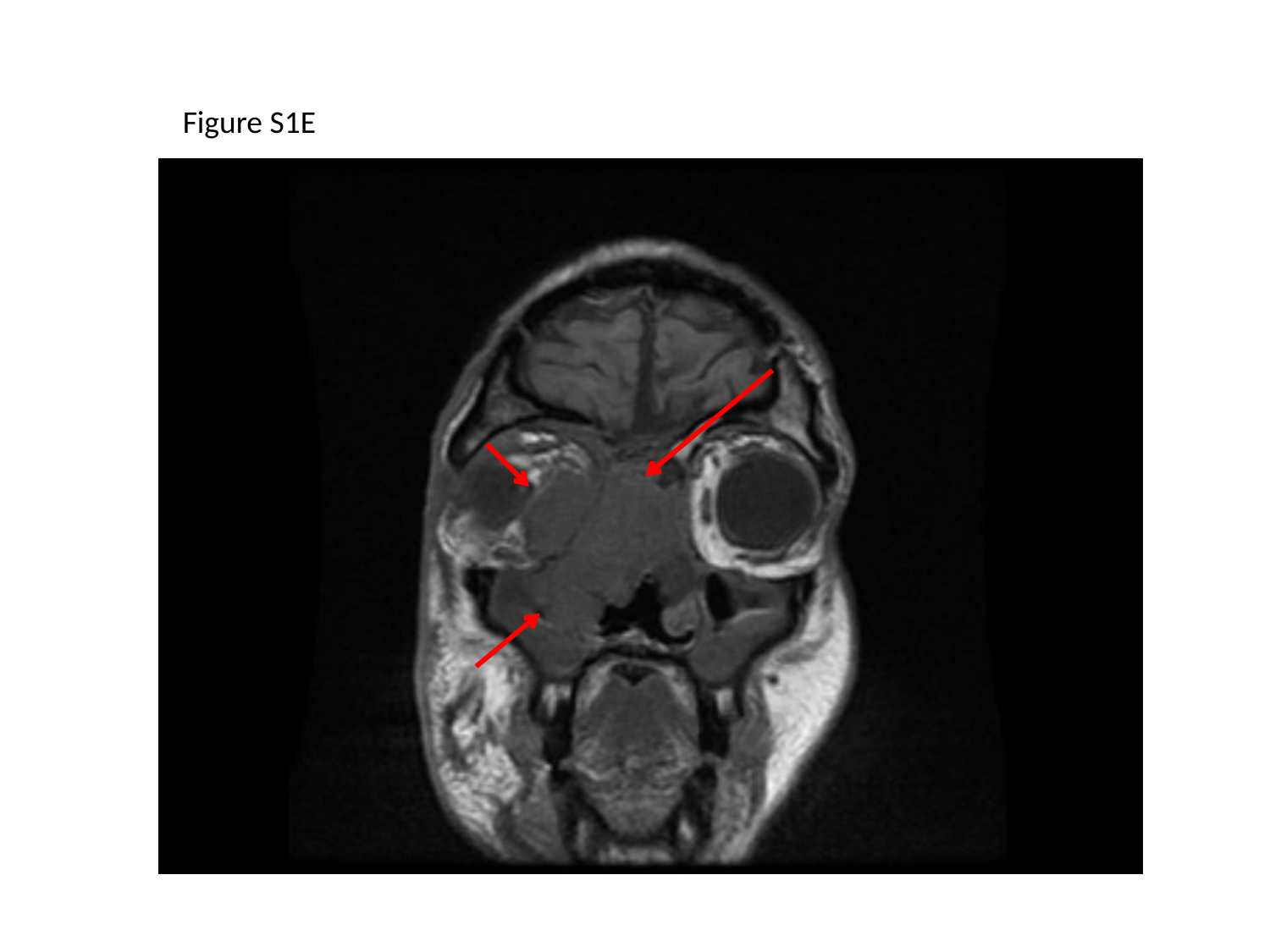

Figure S1E

## Slide 6
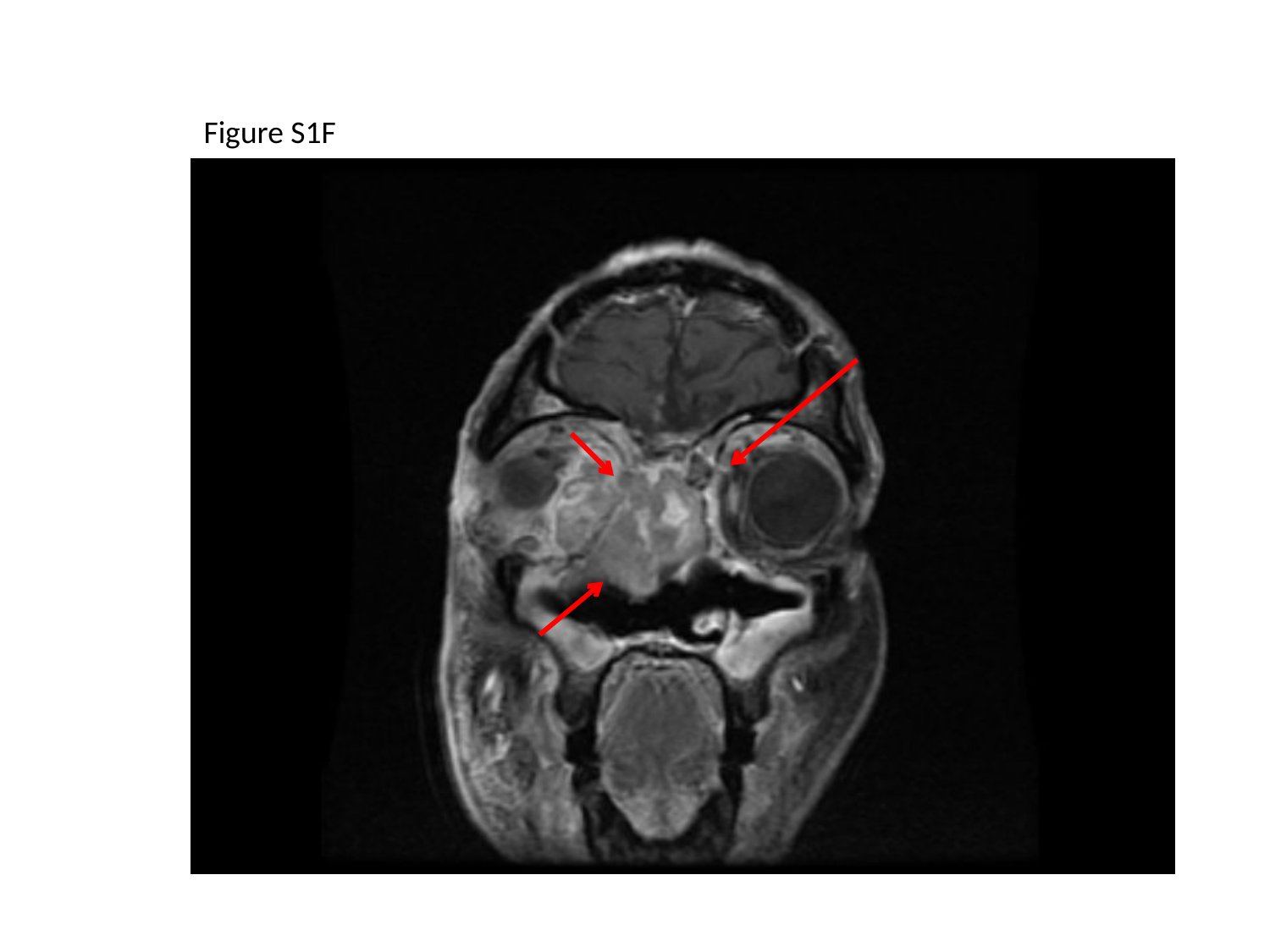

Figure S1F

## Slide 7
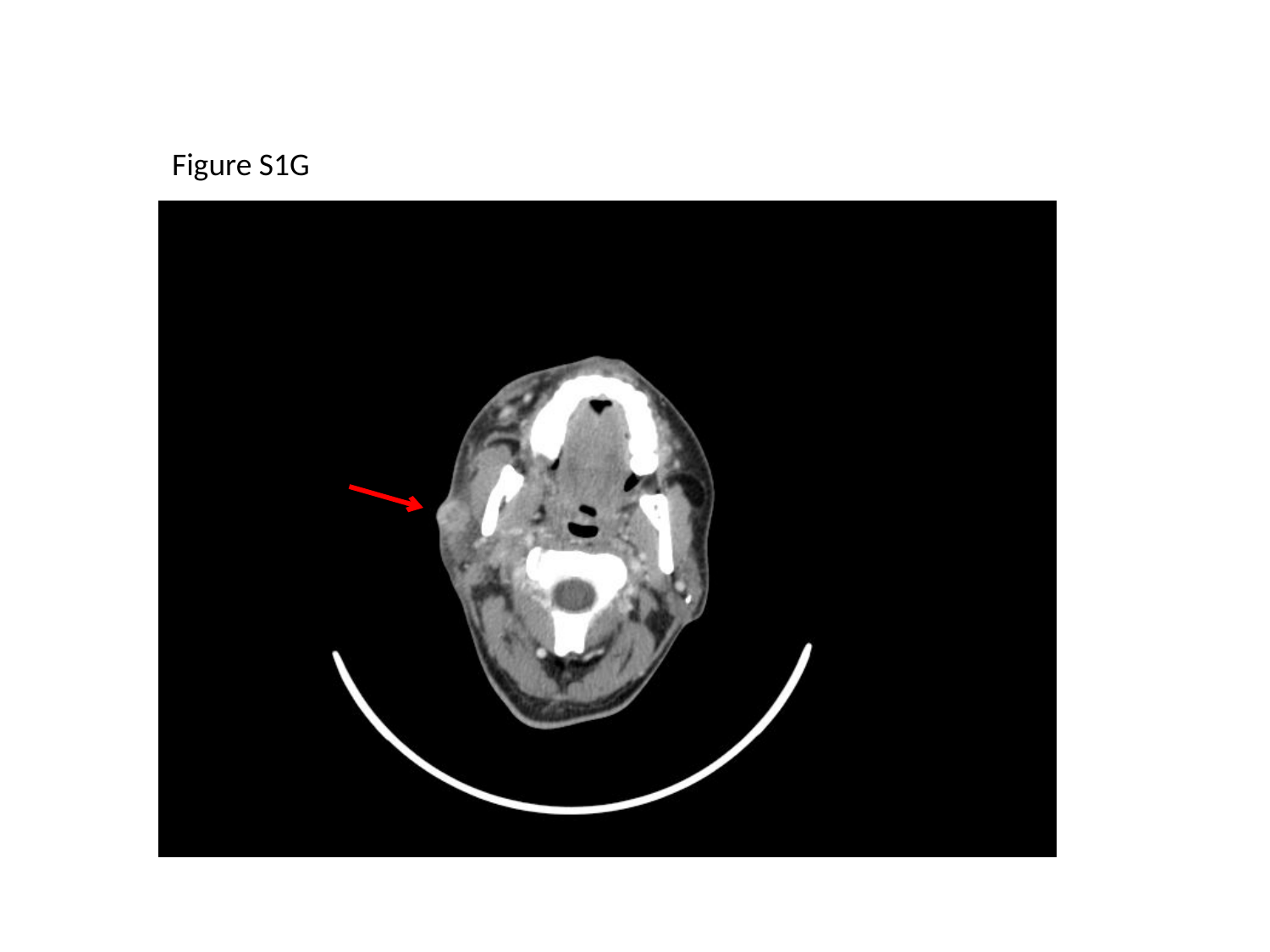

Figure S1G

## Slide 8
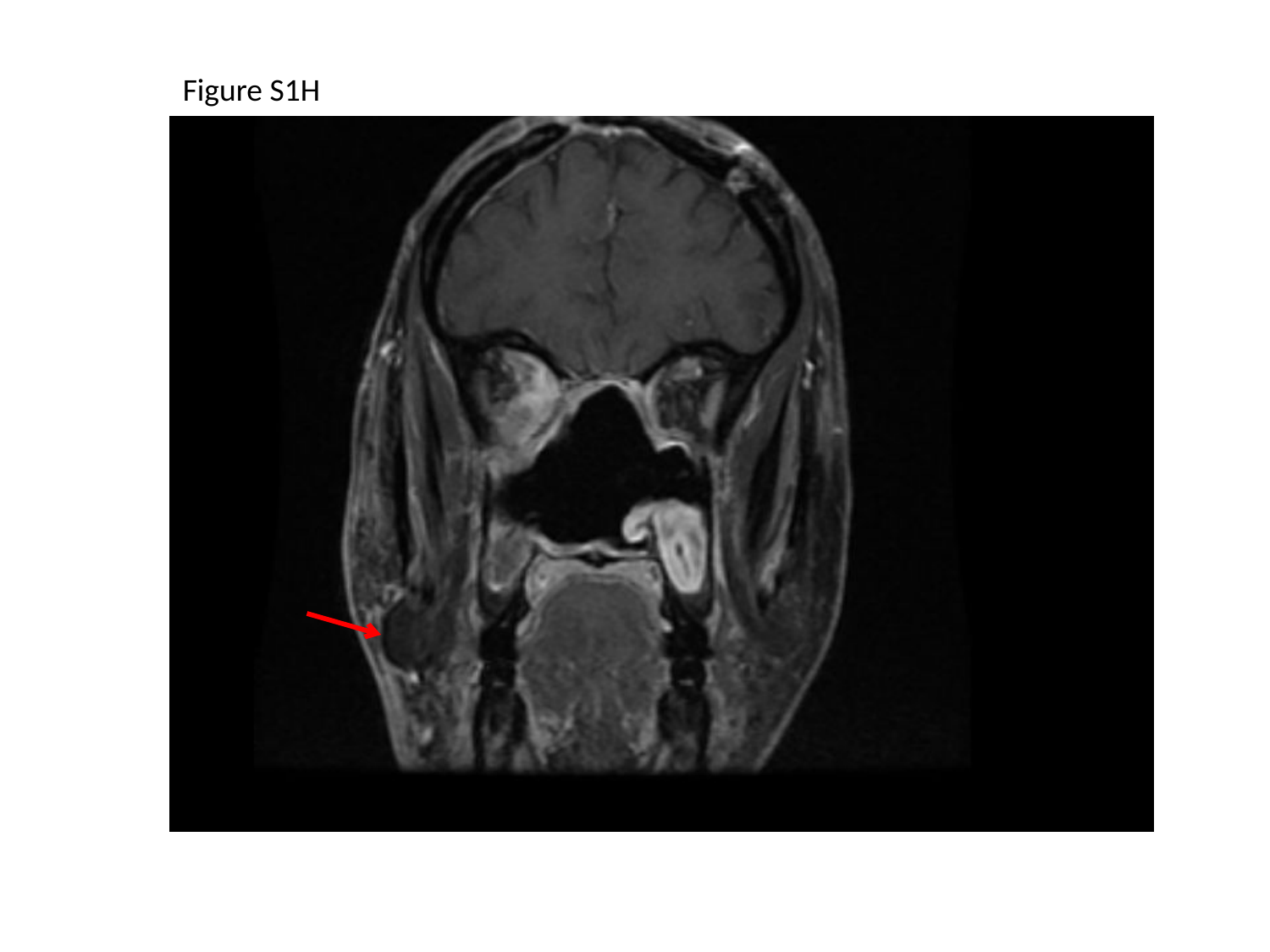

Figure S1H
