# Supplementary figures and images for: Paired Tumor and Normal Whole Genome Sequencing of Metastatic Olfactory Neuroblastoma
Source: PLoS One. 2012 May 23;7(5):e37029. doi: 10.1371/journal.pone.0037029 (PMC3359355; doi:10.1371/journal.pone.0037029)

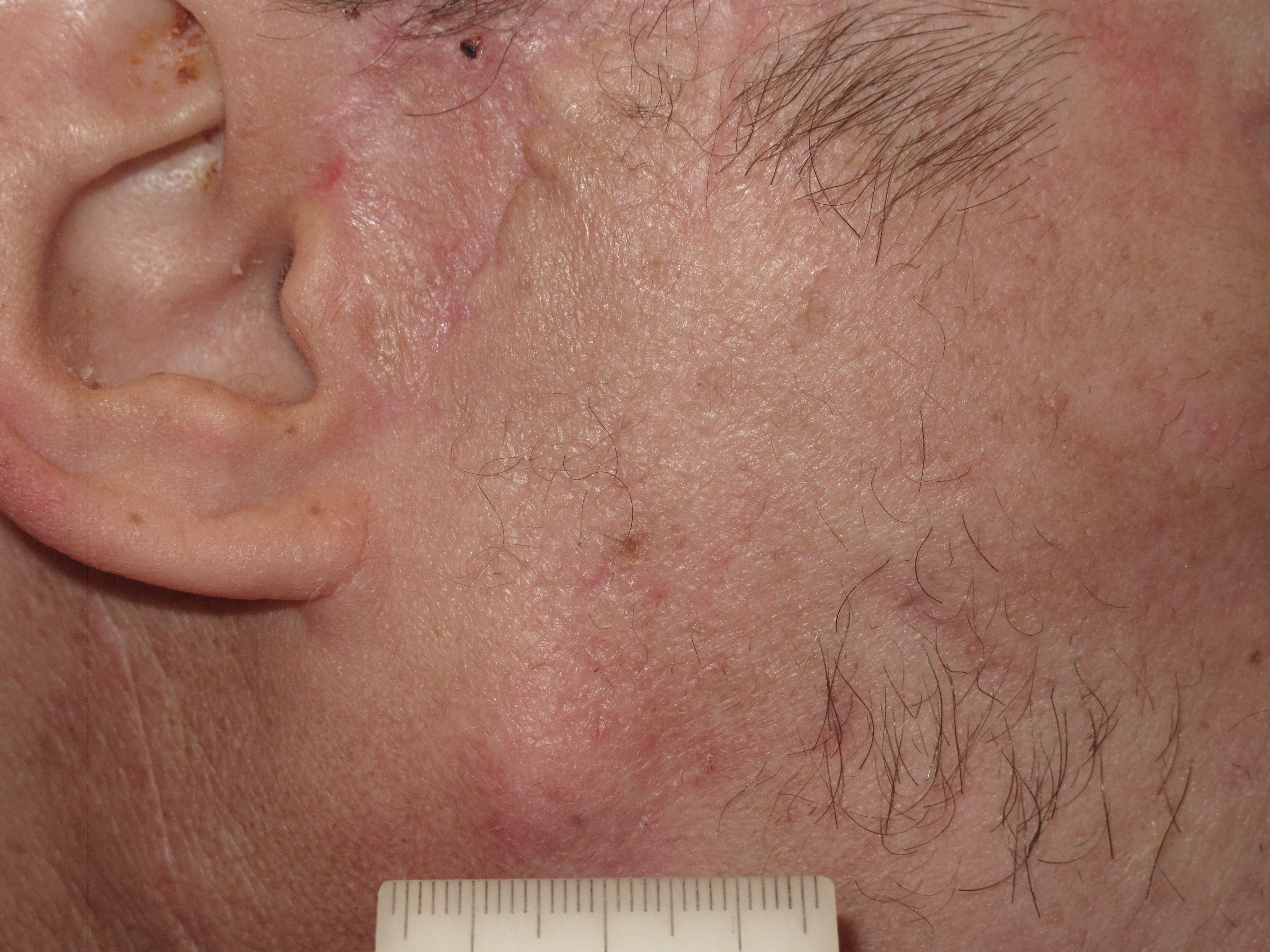

Supplement: Figure S1A — Photograph of metastatic olfactory neuroblastoma mass in the right parotid region that was subsequently biopsied for tumor whole genome sequencing. (TIF) [file pone.0037029.s002.tif]

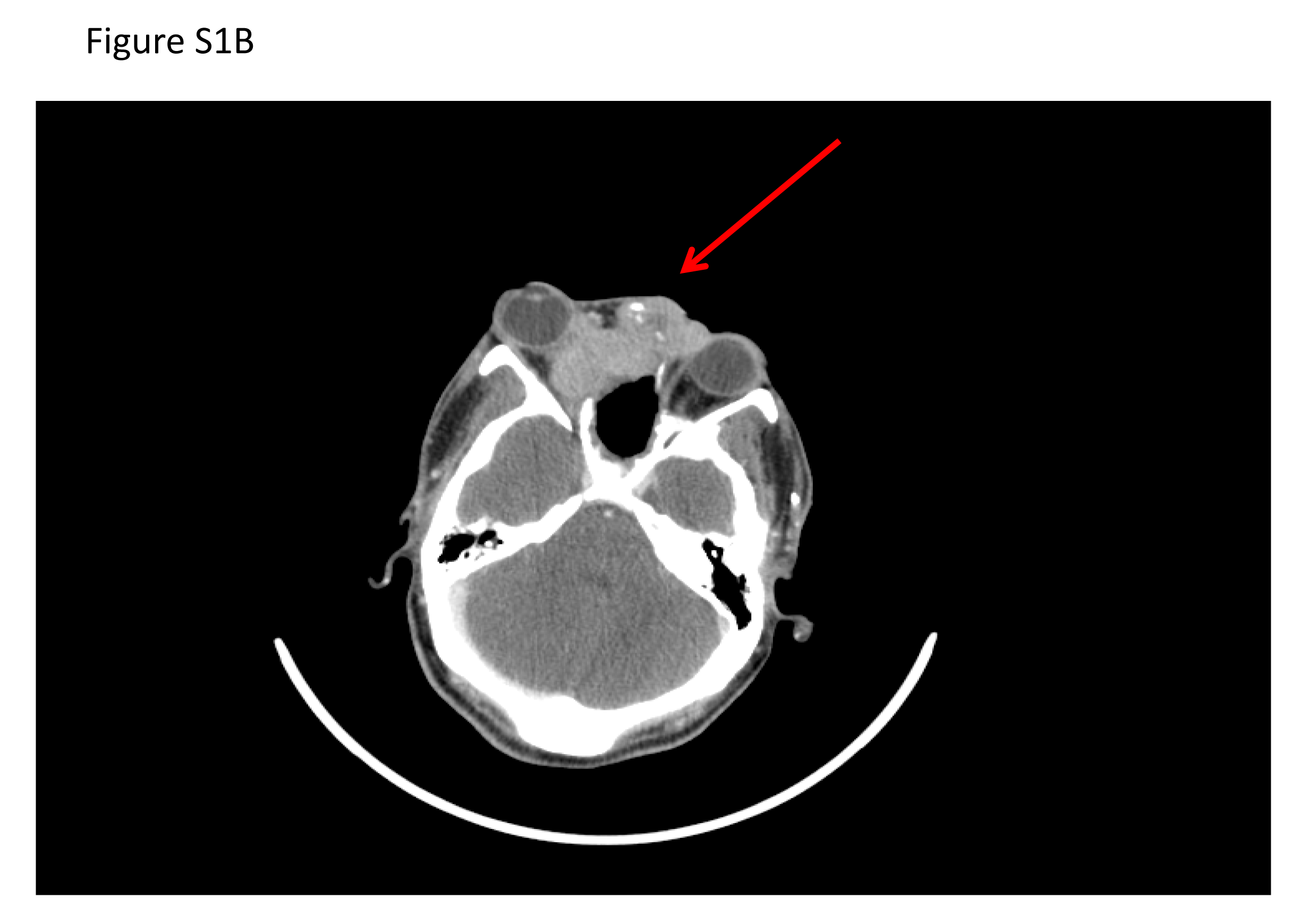

Supplement: Figure S1B — CT axial image depicting extent of local disease recurrence of the olfactory neuroblastoma. Arrow points to the heterogeneous mass. (TIF) [file pone.0037029.s003.tif]

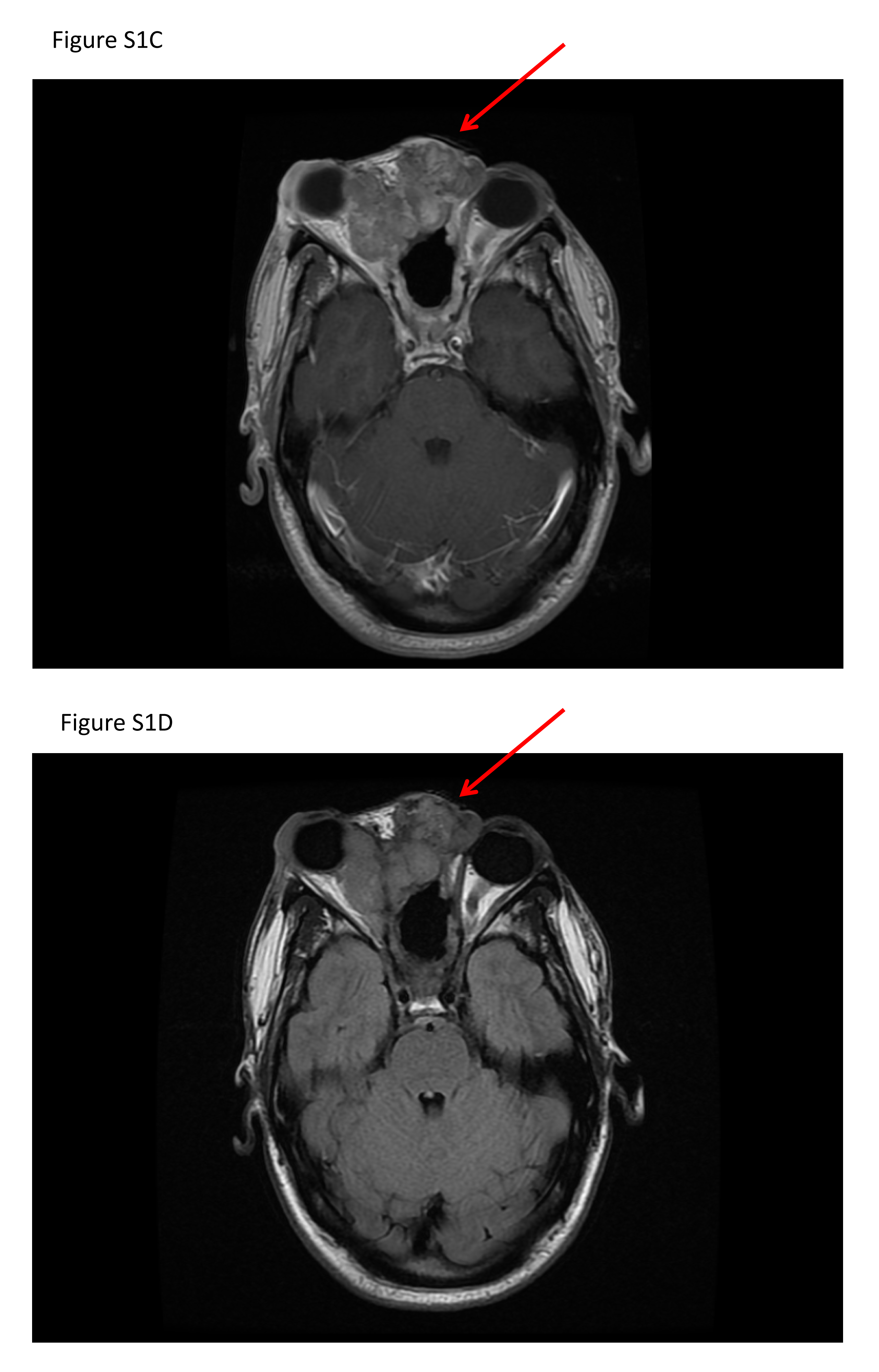

Supplement: Figure S1C and S1D — Spin echo T1 weighted with contrast (1C) and FLAIR sequence (1D) MRI axial images depicting extent of local disease recurrence of the olfactory neuroblastoma. Arrow points to the heterogeneous mass. (TIF) [file pone.0037029.s004.tif]

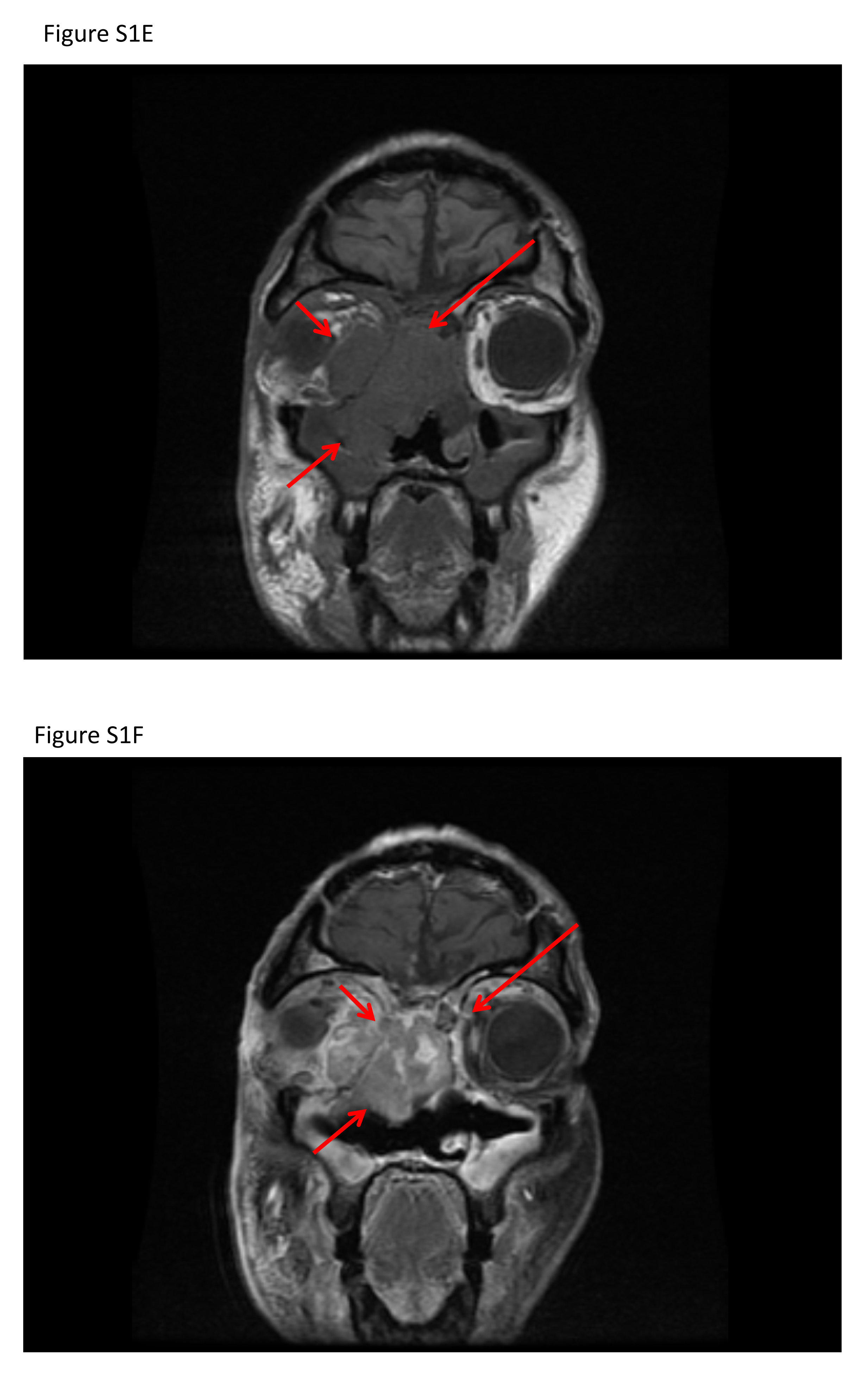

Supplement: Figure S1E and S1F — Spin echo T1 weighted pre-contrast (1E) and spin echo fast scan (1F) MRI coronal images depicting extent of local disease recurrence of the olfactory neuroblastoma. Arrows point to the heterogeneous mass. (TIF) [file pone.0037029.s005.tif]

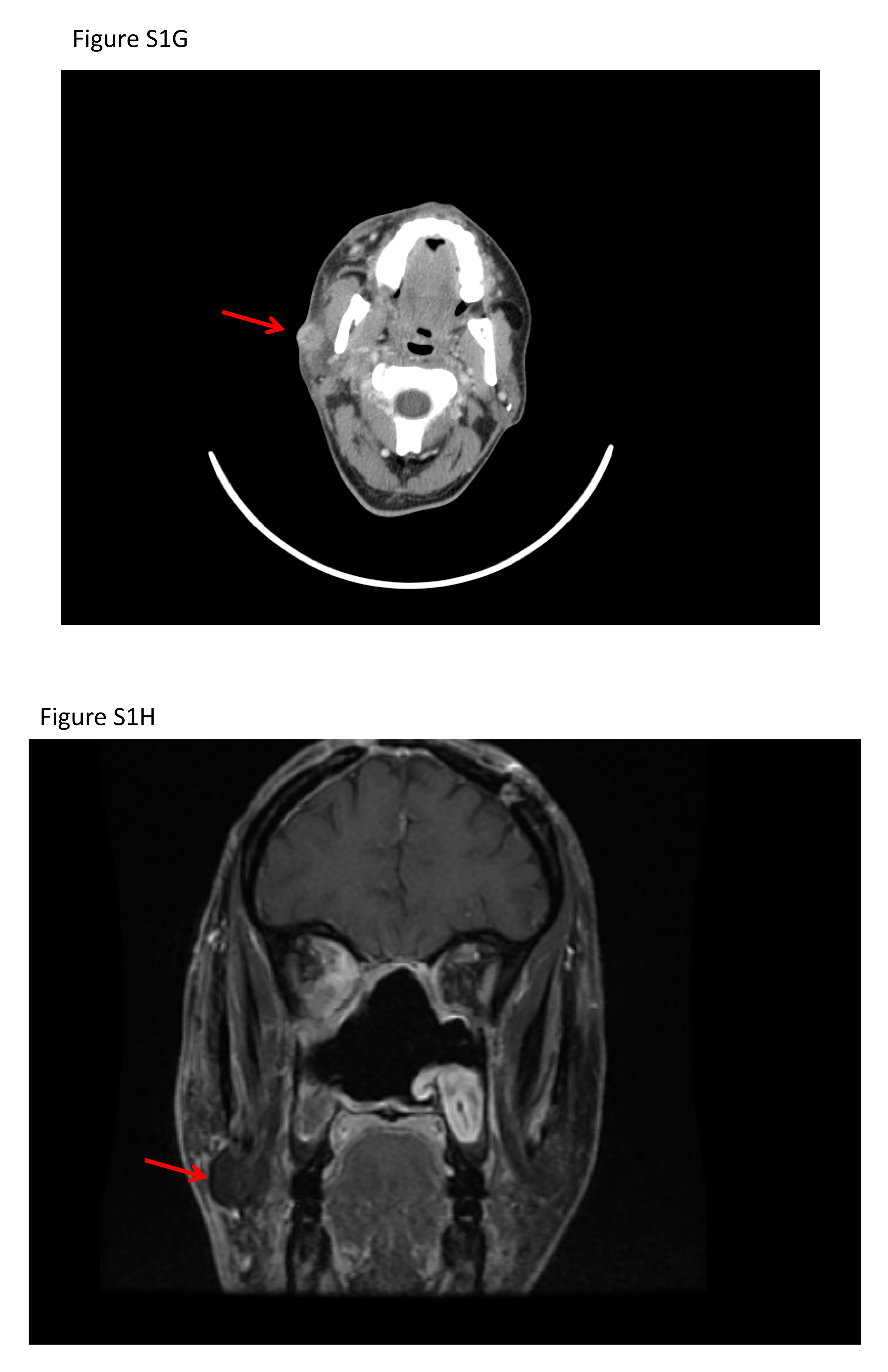

Supplement: Figure S1G and S1H — CT axial image (1G) and MRI coronal spin echo fast scan image (1H) depicting metastasis to the right parotid region. Arrow points to the mass that was biopsied for tumor whole genome sequencing. (TIF) [file pone.0037029.s006.tif]

## Supplementary Figure 2

**A**

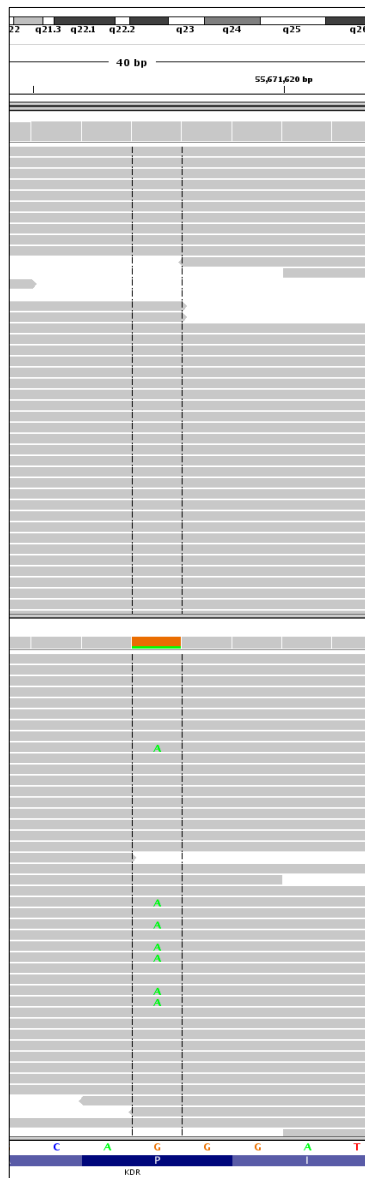

**C**

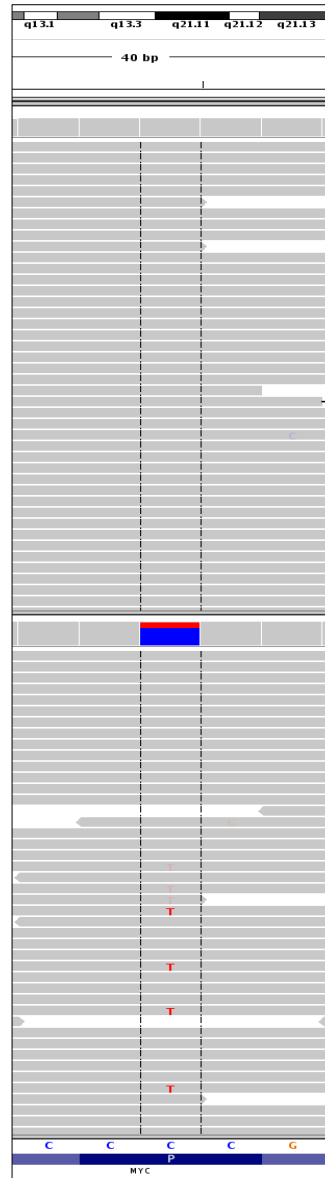

**B**

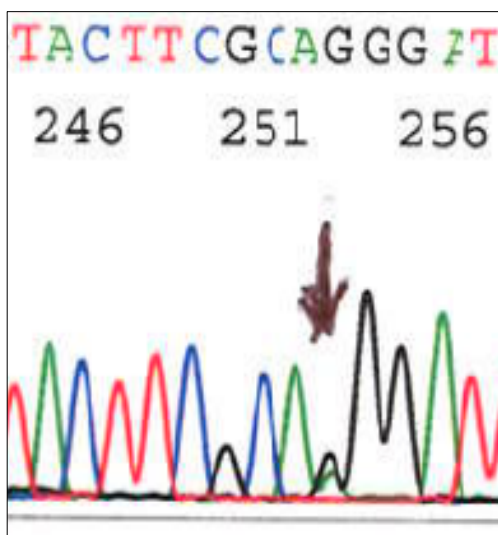

**D**

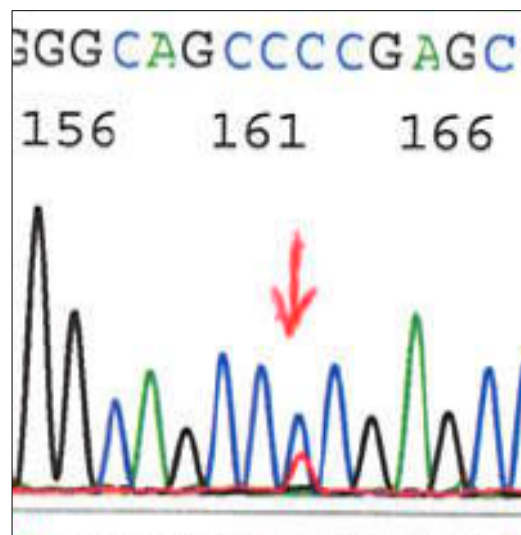

Supplement: Figure S2 — Genomic regions of KDR and MYC genes harboring SNVs. DNA alignments and sequencing electropherograms depicting specific SNVs in KDR (Figures S2A and S2B) and MYC (Figures S2C and S2D) genes. Arrows in Figures S2B and S2D point to the mutated residue in the electropherograms. Electropherogram shown for KDR (Figure S2B) is for the sequencing reaction of the complementary strand. (PDF) [file pone.0037029.s007.pdf]
